# Using weighted regression model for estimating cohort effect in age-period contingency table data

**DOI:** 10.18632/oncotarget.24868

**Published:** 2018-04-13

**Authors:** I-Shiang Tzeng, Chau Yee Ng, Jau-Yuan Chen, Li-Shya Chen, Chin-Chieh Wu

**Affiliations:** ^1^ Department of Research, Taipei Tzu Chi Hospital, Buddhist Tzu Chi Medical Foundation, New Taipei City, Taiwan; ^2^ Department of Statistics, National Taipei University, Taipei, Taiwan; ^3^ Bachelor's Program of Financial Engineering and Actuarial Science, Feng Chia University, Taichung City, Taiwan; ^4^ Department of Dermatology, Drug Hypersensitivity Clinical and Research Center, Chang Gung Memorial Hospital, Taipei, Taiwan; ^5^ School of Medicine, College of Medicine, Chang Gung University, Taoyuan, Taiwan; ^6^ Department of Family Medicine, Chang-Gung Memorial Hospital, Linkou Branch, Taoyuan City, Taiwan; ^7^ College of Medicine, Chang Gung University, Taoyuan, Taiwan; ^8^ Department of Statistics, National Chengchi University, Taipei, Taiwan; ^9^ Department of Emergency Medicine, Chang Gung Memorial Hospital, Keelung, Taiwan

**Keywords:** age-period-cohort, multiphase method, prediction, hepatocellular carcinoma

## Abstract

**Background:**

Recently, the multiphase method was proposed to estimate cohort effects after removing the effects of age and period in age-period contingency table data. Hepatocellular carcinoma (HCC) is the most common primary malignancy of the liver and is strongly associated with cirrhosis, due to both alcohol and viral etiologies. In epidemiology, age-period-cohort (APC) model can be used to describe (or predict) the secular trend in HCC mortality.

**Results:**

The confidence interval (CI) of the weighted estimates was found to be relatively narrow (compared to unweighted estimates). Moreover, for males, the mortality trend reverses itself during 2006–2010 was found from an increasing trend into a slightly deceasing trend. For females, the increasing trend reverses (earlier than males) itself during 2001–2005.

**Conclusions:**

The weighted estimation of the regression model is recommended for the multiphase method in estimating the cohort effects in age-period contingency table data.

**Impact:**

The regression model can be modified through the weighted average estimate of the effects with narrower CI of each cohort.

**Methods:**

After isolating the residuals during the median polish phase, the final phase is to estimate the magnitude of the cohort effects using the regression model of these residuals on the cohort category with the weight equal to the occupied proportion according to the number of death of HCC in each cohort.

## INTRODUCTION

Evaluating disease and mortality patterns over time has become popular in understanding the utility of disease etiology in public health. However, the trend assessment of age-specific mortality presented inconsistent patterns between age groups. Birth cohort analyses are valuable in predicting future increases (or decreases) of diseases under the same pattern among birth cohorts. In epidemiology, one popular interpretation on the relationship between age, period, and cohort (APC) variables is that age and period interact to create unique generational experiences. Age effects are correlated with the outcome at various ages, such as deaths caused by cancer. Simultaneously, period effects influenced all ages over time. Birth cohort effects presented changes across groups with the same birth year who had the same outcome during the same period. Disease mortality is not only influenced by birth cohort effects but also affected by age and period. For example, if a person born in 1980 (i.e., birth cohort effects) is highly at risk of dying due to cardiovascular disease during his/her lifetime, it will take at least 30 years (i.e., period effects) for him/her to die during adulthood (i.e., age effects) at the beginning of 2010. Therefore, the conceptualization of cohort effects was proposed based on the interaction between age and period [1]. Although this conceptualization still has an exact linear relationship (age + cohort = period), exposures (predictors) are not intrinsic to birth cohorts. We would rather explain a cohort effect that existed while different disease distributions arise. However, as age + cohort = period, these three variables are linear dependent, and unless additional constraints are imposed, APC model that estimates the linear effects of age, period, and cohort is non-identifiable. We have explained this problem and the potential constraints imposed in our previous publications [2–5]. However, methodological complexity is a barrier for many researchers. As previously mentioned, a cohort effect is conceptualized as a period effect that is differentially experienced through age-specific exposure to an event or cause (i.e., interaction) [6]. Addressing the identifiable problem in this conceptualization is unnecessary because cohort effects are not conceptualized independently from age and period. The median polish analysis has been used to estimate cohort effects under this conceptualization [6, 7].

Recently, the multiphase method was proposed by Keys and Li [6] and provides three phases of estimating cohort effects with minimal assumptions on the contingency table data. Moreover, the median polish does not rely on a specific distribution or structure and thus can be widely applied to various types of data, such as rates, log rates, proportions, and counts. The first phase is graphical representation. Graphs were conducted by age across periods or birth cohorts and even birth cohort across ages or periods. For example, we conduct a graph of rates of age across periods. If age-specific rates of different age groups varied mutually among different periods, then the period effect may exist in contingency table data. Cohort effect can also be present while age-specific rates of different age groups interacted mutually among different periods. The second phase involves median polish analysis to remove the additive effect of age and period by iteratively subtracting the median from each row and column. The final phase is regression procedure, which contain cohort effects and random error. We regressed these residuals on the cohort category (defined as an indicator variable) in a linear regression model with the aggregated count data in the format of contingency tables.

The median polish was developed to describe data in a two-way contingency table [8] and remove the additive influence of age (i.e., row) and period (i.e., column) by iteratively subtracting the median from each row and column. Selvin first applied the median polish to APC analysis [7]. This technique requires no assumptions about the distribution or structure of the data in a two-way contingency table. Consequently, it can be widely used for any type of data contained in a table without any assumption, such as suicide data [9]. APC model was also used to describe the secular trend in disease incidence or mortality [3]. The APC model usually assumed that age, period, and cohort have additive effects on the log transformation of disease/mortality rate.

Hepatocellular carcinoma (HCC) is the most common primary malignancy of the liver and is strongly associated with cirrhosis, due to both alcohol and viral etiologies [10]. Of all malignant tumors worldwide, HCC ranked fifth in terms of mortality in men (and the eighth in women). In Taiwan, it has been ranked as the first among all major cancers in men (and second in women) [11].

In this study, we investigated the longitudinal trends of HCC mortality data from the Vital Statistics as our demonstration. We evaluated the HCC mortality to identify the effects of age, period, and cohort and examined whether these effects varied by gender. This study aimed to use weighted average method to modify the multiphase method, in order to estimate the cohort effect. We also illustrated how to estimate the cohort effect using the multiphase method and compared the results to those estimated by proposed weighted average method.

## RESULTS

**Figure 1:**
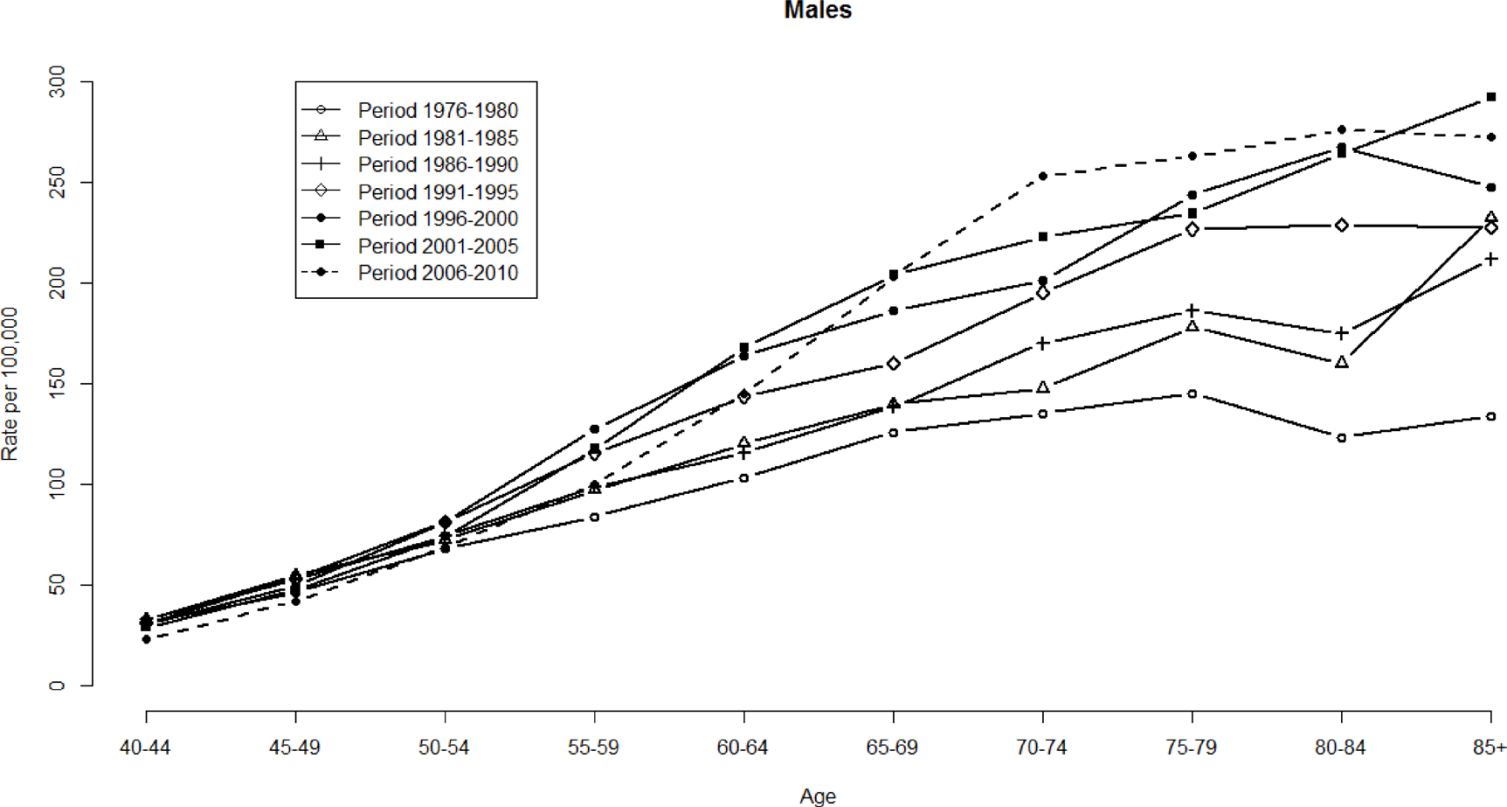
HCC mortality rates per 100,000 by age and period, males, Taiwan, 1976–2010

**Figure 2:**
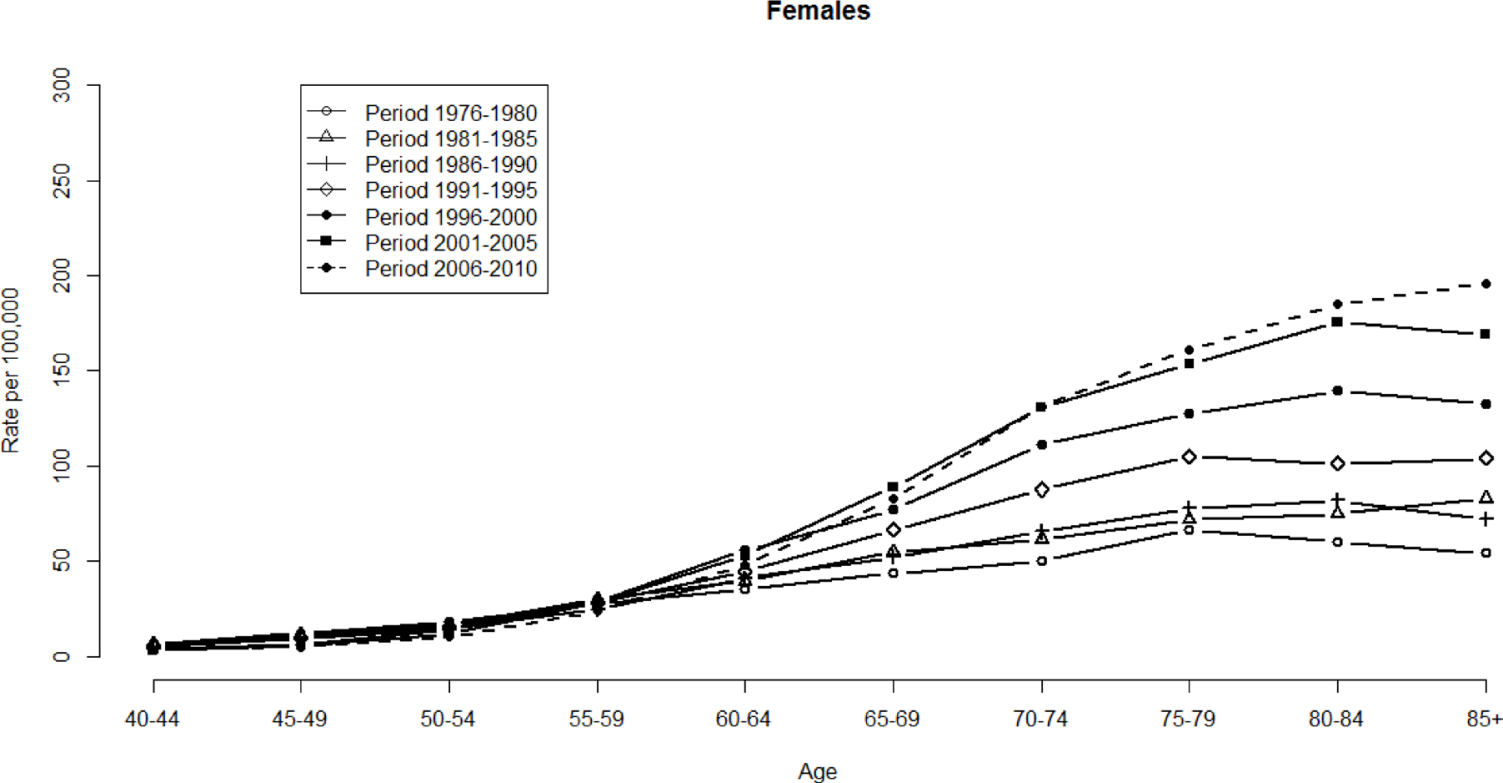
HCC mortality rates per 100,000 by age and period, females, Taiwan, 1976–2010

Figures 1 and 2 show the HCC mortality rates among age and period groups. These fluctuations were more significant among men than women. The distribution of rates according to age shows that HCC mortality rates begin at 40–44 age group (see Figure 1). Note that HCC mortality rates rose gradually among those in ≥60 age group (see Figure 2). However, HCC mortality rates based on age have considerably changed over time, which means that a significant cohort effect was hidden in the usual age-period cross-classified Vital Statistics table and will not apparent until the distant future. We perform the median polish procedure on the log-transformed HCC mortality rates. Tables 1 and 2 present the estimated cohort effects of the APC model on HCC mortality rates. Moreover, Table 3 also presents the age and period effects for both gender. Subsequently, Tables 1 and 2 report the weighted estimates obtained after calculating the weighted average procedure for both gender. According to the smallest deviance (compared to unweighted estimates) of confidence interval (CI) of the weighted estimates, the weighted estimates are better to fit the data. For men, in the left panel of Table 1 presents the cohort effects of the birth cohorts. The cohort effect increases from 0.75 (the earliest cohort effect in 1891) to 1.13 (the greatest cohort effect in 1936). For women, the cohort effect increases from 0.69 (the earliest cohort effect in 1891) to 1.17 (the greatest cohort effect in 1926). Note that the cohort effect significantly increased by approximately 51% and 68% compared to the cohort in 1891 for men and women, respectively. In the right panel of Table 1, the increase was evenly distributed. Here, the cohort effect increased from 0.71 (the earliest cohort effect in 1891) to 1.05 (the greatest cohort effect in 1936). For women, the increased distribution is presented similarly in the right panel of Table 2. The cohort effect increased from 0.65 (the earliest cohort effect in 1891) to 1.04 (the greatest cohort effect in 1921). Thus, we observed that the mortality rate increasing by approximately 48% and 60% will become the peak value for men and women, respectively.

**Table 1:** Estimated rate ratios and 95% conference intervals for effect of birth cohort on hepatocellular carcinoma mortality of males in Taiwan, 1891–1966

	Unweighted		Weighted	
	Effects	95% CI for Effects	Effects	95% CI for Effects
Cohort				
(1891∼1966)				
1891	0.75	0.62–0.89	0.71	0.59–0.84
1896	0.90	0.82–0.99	0.86	0.79–0.94
1901	0.91	0.85–0.97	0.81	0.73–0.90
1906	0.93	0.88–0.98	0.85	0.79–0.92
1911	0.97	0.92–1.02	0.89	0.84–0.94
1916	1.03	0.98–1.08	0.98	0.95–1.01
1921	1.01	0.97–1.06	0.98	0.96–1.00
1926	1.01	0.97–1.05	0.99	0.97–1.01
1931	1.06	1.01–1.10	1.03	1.01–1.05
1936	1.13	1.09–1.18	1.05	1.04–1.07
1941	1.09	1.04–1.14	1.04	1.03–1.06
1946	1.00	REF	1.00	REF
1951	0.87	0.82–0.92	0.93	0.91–0.95
1956	0.82	0.77–0.88	0.87	0.85–0.90
1961	0.77	0.70–0.85	0.74	0.70–0.78
1966	0.68	0.57–0.82	0.79	0.76–0.82

Note: REF = reference; CI = confidence interval.

**Table 2:** Estimated rate ratios and 95% conference intervals for effect of birth cohort on hepatocellular carcinoma mortality of females in Taiwan, 1891–1966

	Unweighted		Weighted	
	Effects	95% CI for Effects	Effects	95% CI for Effects
Cohort				
(1891∼1966)				
1891	0.69	0.44–1.10	0.65	0.41–1.03
1896	0.84	0.66–1.07	0.78	0.61–1.00
1901	0.82	0.68–0.97	0.73	0.56–0.95
1906	0.85	0.74–0.97	0.78	0.68–0.89
1911	0.89	0.79–1.00	0.86	0.79–0.93
1916	1.01	0.90–1.12	0.98	0.94–1.02
1921	1.11	1.01–1.23	1.04	1.01–1.07
1926	1.17	1.06–1.30	1.03	1.01–1.05
1931	1.13	1.02–1.25	1.02	1.00–1.03
1936	1.16	1.05–1.28	1.02	1.00–1.03
1941	1	REF	1	REF
1946	0.86	0.77–0.97	0.97	0.95–0.99
1951	0.67	0.58–0.77	0.86	0.84–0.89
1956	0.74	0.62–0.88	0.79	0.75–0.83
1961	0.42	0.33–0.54	0.52	0.47–0.57
1966	0.49	0.31–0.78	0.49	0.44–0.54

Note: REF = reference; CI = confidence interval.

**Table 3:** Estimated age and period effects of among males and females, Taiwan, 1976–2010

	Males	Females
	Effect	Effect
Period		
1976–1980	–0.25	–0.32
1981–1985	–0.10	–0.15
1986–1990	–0.08	–0.16
1991–1995	0.04	0.02
1996–2000	0.11	0.20
2001–2005	0.16	0.25
2006–2010	0.13	0.16
Age		
40–44	–1.37	–2.01
45–49	–0.83	–1.36
50–54	–0.41	–0.90
55–59	–0.09	–0.29
60–64	0.12	0.20
65–69	0.32	0.53
70–74	0.45	0.83
75–79	0.54	1.02
80–84	0.62	0.98
85+	0.67	1.00
Constant	–6.74	–7.89

**Figure 3:**
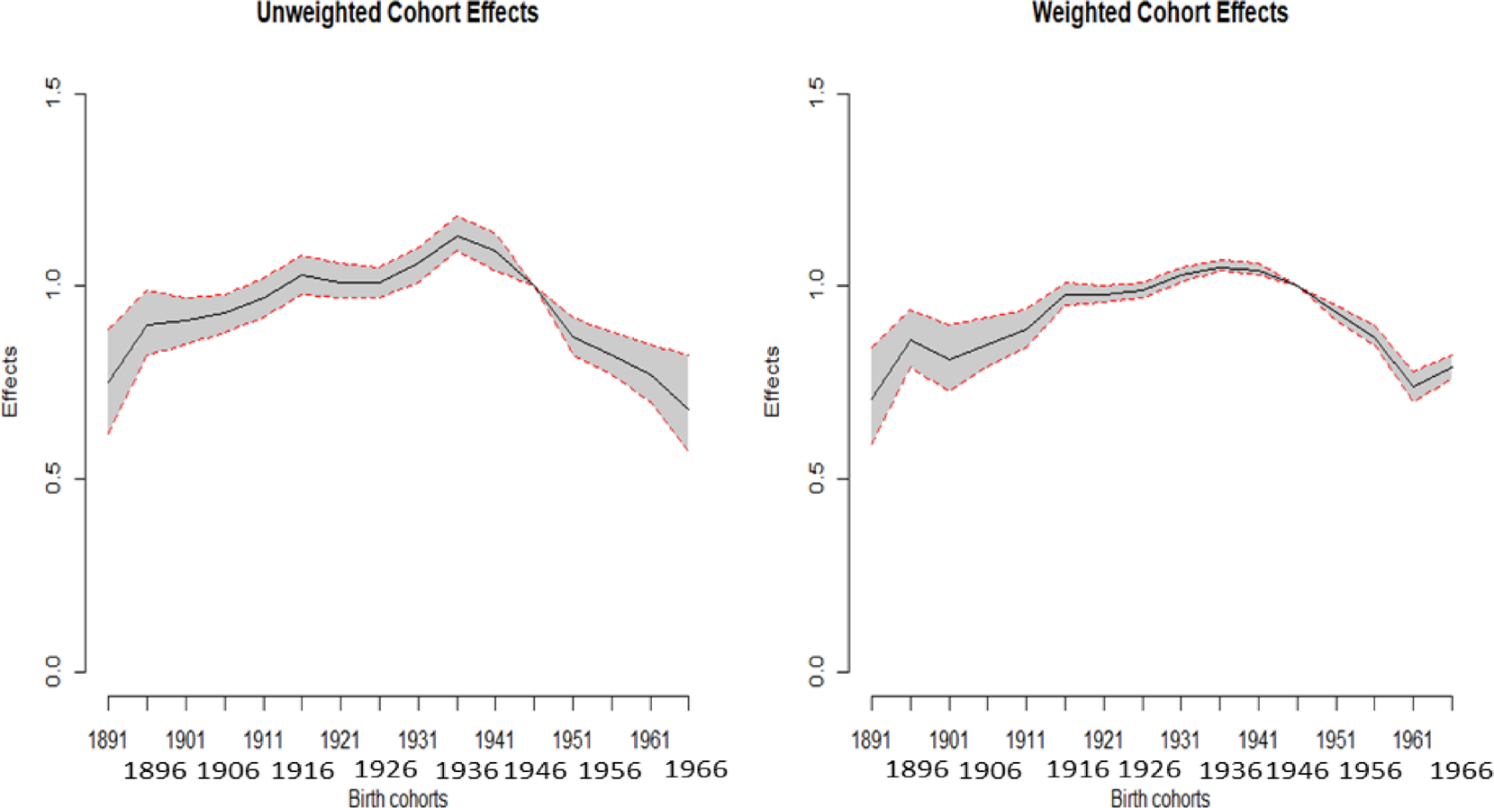
Plot of the unweighted and weighted effects with 95% confidence interval of males

**Figure 4:**
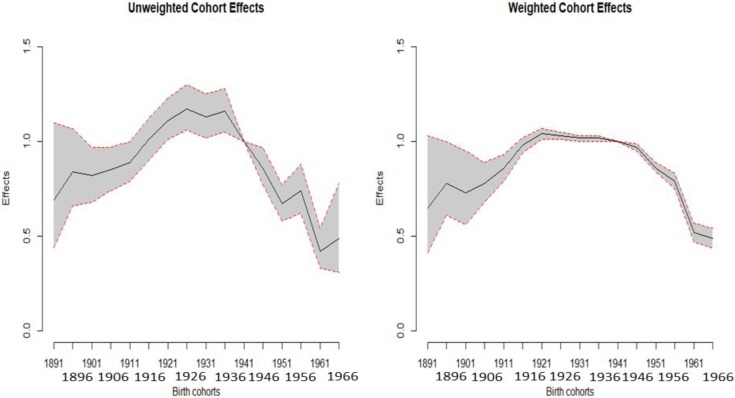
Plot of the unweighted and weighted effects with 95% confidence interval of females

Among the birth cohorts, men born in 1936 exhibited the highest risk of HCC mortality (Table 1). Consequently, for weighted estimates, the effect was 1.05 (95% CI: 1.04–1.07) for the 1936 birth cohort compared to the reference birth cohort in 1946. However, a dramatically decreasing trend was observed for the earlier cohorts. Additionally, the effects were reversed after the 1936 cohort. Moreover, we plot the unweighted and weighted cohort effects with 95% CI of men and women (Figures 3 and 4), respectively. Both figures show that almost all of the widths of 95% CI of weighted are shorter than that of unweighted cohort effects.

In this study, we limited our APC analysis of the median polish procedure to estimating cohort effects and 95% CIs of the HCC mortality. Based on this analysis, it appears that the residual errors (*Ɛ_ijk_*) were close to zero.

## DISCUSSION

**Figure 5:**
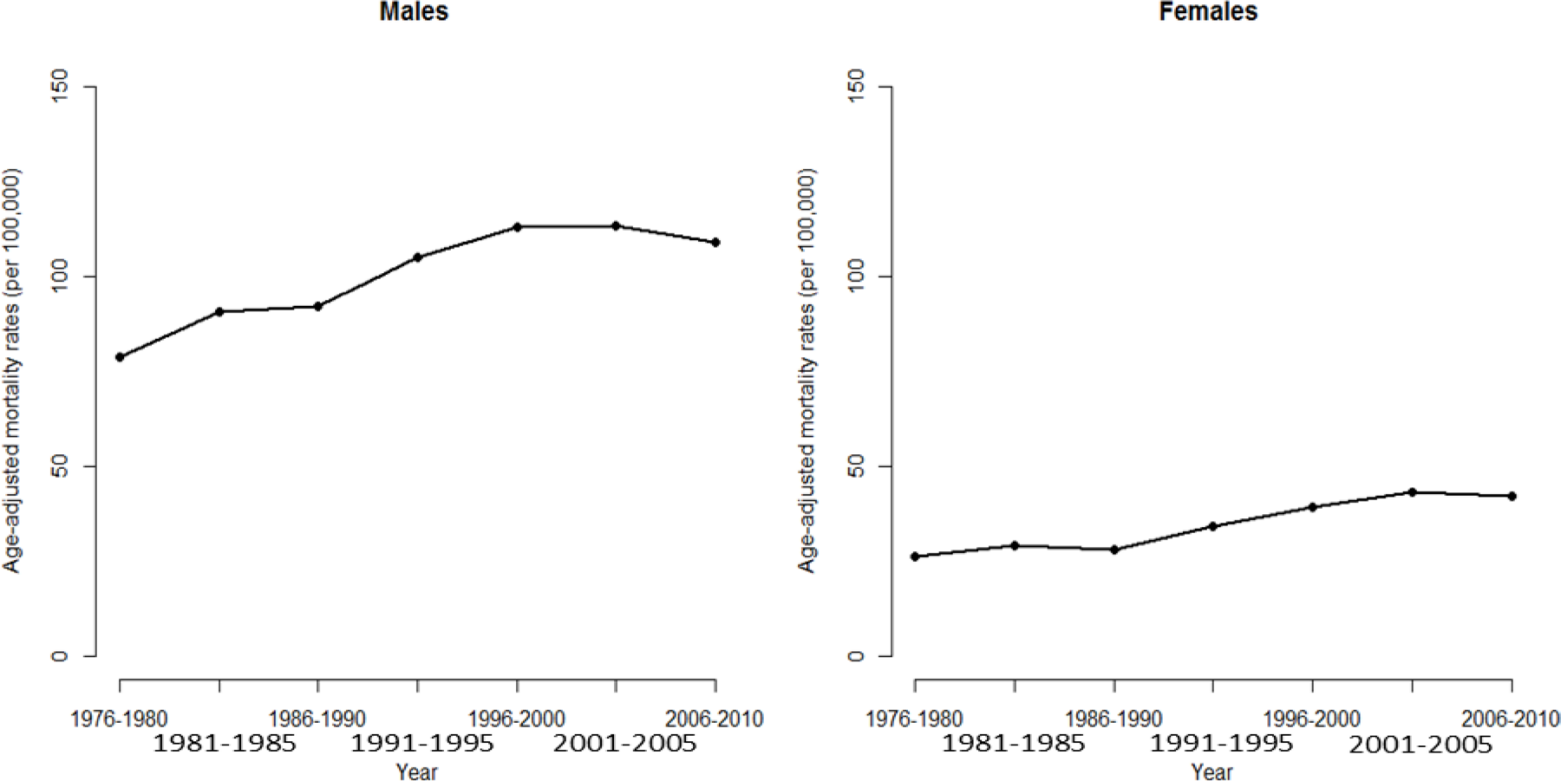
Age-adjusted mortality rate of death from hepatocellular carcinoma for men and women in Taiwan

Considering the time trend of HCC mortality, the conventional analysis using a simple linear extrapolation of the observed log age-adjusted rates may underestimate some important characteristics hidden in the data (such as the cohort effects) and facilitate prediction that are grossly missing. If we directly observe the long-term trends of HCC mortalities from 1976 to 2010 in Taiwan (Figure 5), no one with any reason will doubt that the trends, having been increasing for 35 years, will increase for the next few years. However, in fact the recent trend on HCC mortalities in Taiwan is decreasing and is driven by cohort effects (identified from the APC analysis), which as described decreased after the 1936 cohort. In this study, applying APC model allows advanced and more accurate warning for trend changes.

From a clinical viewpoint, hepatitis B virus (HBV) infection is an important health issue worldwide with high morbidity, approximately 2 billion people infected and 350 million suffering from chronic HBV infection [12]. The HBV infection can induce a wide range of clinical problems, from inactive carrier status to fulminate hepatitis, cirrhosis, or hepatocellular carcinoma. Injecting hepatitis B vaccine is the most effective prevention method. Based on the policy implications, the first worldwide hepatitis B mass vaccination program was implemented in 1984 in Taiwan [13]. They screened pregnant women for HBsAg and then HBeAg. At first, the immunization program covered only infants of HBsAg carrier mothers in initially 2 years. From the third year of vaccination program, all infants were covered. Recently, the coverage rate of hepatitis B vaccine reached 99%. After three vaccines, approximately 90–95% of the people will have life-long immunity. Note that the decline in pediatric HCC in Taiwan can be attributed to the contribution of this worldwide vaccination program. The APC estimation described in this paper can have an advanced warning for these (increased) trend changes (to be decreased recently).

This study investigated trend of cohort effect through applying median polish procedure. The weighted estimates for modification of the regression model of these residuals then allow a weighted average estimate on the effect with narrower CI of each cohort. The results are reported in the form of cohort effects using the 1946 cohort, because these categories present the fewest changes in HCC mortality rates with cohort influence removed.

In most modeling methods (such as linear or nonlinear regression models), one of the common assumptions is that each real value of data provides equal information to estimate the parameters in a model which was undertaken. It means that the standard deviation of the error term is the constant underlying predictor variables. Based on our literature reviews, the assumption does not hold in modeling to empirically estimate the parameters. When we use weighted regression, the unknown parameters are estimated, a less weight is given for the less precise data points and more weights are given for the precise data points. The advantage is that weighted procedure can reduce the standard deviation of the estimator. However, the drawback of the weighted regression method is almost unknown in empirical practice. Because the exact weight is almost unknown, the estimated weight can be used to estimate the parameters. Moreover, experience shows that the weighting due to estimation does not change much and often does not affect regression analysis or its interpretation [14]. Theoretically, any disease with rates governed by age, period, and cohort effects is amenable for an APC model. Moreover, the weighted average estimates can be used for prediction [15–17]. If the CI is relatively narrow, the uncertainty is smaller, because the CI describes the uncertainty inherent in this estimate and range of values within which we can be reasonably sure that the true effect actually happens.

Several potential limitations of our study should be noted. First, we can only infer about the etiologies of the changes observed. The HCC mortality based on age, period, and cohort effects are re-amenable for an APC model. However, the presence of set assumptions for the median polish that we used should be noted in the present study. Second, APC analysis can be used extensively in the epidemiology field in populations of developing or recently developed countries, where long-running cohort studies are limited. Third, we do not have information from the aggregated format datasets to adjust confounders, such as comorbidities or lifestyle, in the APC model. Further studies using individual data is needed to solve this limitation. Fourth, we use the number of deaths due to HCC as the weight to modify the regression procedure in the multiphase method. Because the exact weight is almost unknown, the use of various weights may cause minor inflation among estimated cohort effects. Lastly, circumstances in which various APC estimation methods to address the non-identifiable problem may occur (e.g., Holford adopts the linear and curvature trends to tackle the non-identifiable problem [18]). Meanwhile, the median polish provides conceptual shift form complex assumption among APC model to estimate the cohort effect with a minimum of assumptions and easily applies a general format for contingency table.

In conclusion, the weighted estimation to modify the regression model then allows a weighted average effect with narrower CI of each cohort. In summary, the weighted estimation of the regression model is recommended for multiphase method to estimate the cohort effects in age-period contingency table data.

## MATERIALS AND METHODS

### Data source

To illustrate the calculations, we used the HCC mortality data from 1976 to 2010 for men and women in Taiwan, which were obtained from individual health records of the Ministry of Health and Welfare (MOHW). HCC mortality was classified based on the International Classification of Disease (ICD) Code 150. The mortality data were available for 10 five-year age groups (40–44, 45–49, 50–54, 55–59, 60–64, 65–69, 70–74, 75–79, 80–84, and 85+), 7 five–year time periods (1976–1980, 1981–1985, 1986–1990, 1991–1995, 1996–2000, 2001–2005, and 2006–2010), and 16 birth cohorts (mid–cohort years: 1891, 1896, 1901, 1906, 1911, 1916, 1921, 1926, 1931, 1936,1941, 1946, 1951, 1956, 1961, and 1966). In Table 4, we present an age–period contingency table format for the HCC mortality of men and women. From these, we calculated the age–specific and the age–adjusted (using the 2000 World Standard Population) mortality rates [19].

**Table 4:** Age-period contingency table of HCC mortality rate per 100,000 among males and females, Taiwan, 1976–2010

Males	1976–1980	1981–1985	1986–1990	1991–1995	1996–2000	2001–2005	2006–2010
40–44	31.41	33.10	33.20	31.24	30.40	29.10	23.26
45–49	46.50	55.13	52.88	53.61	49.79	47.66	42.25
50–54	68.11	72.47	74.56	81.73	81.65	74.19	69.12
55–59	84.12	97.32	98.96	115.57	127.55	117.70	100.19
60–64	103.58	120.45	115.71	143.74	164.14	168.20	145.35
65–69	126.15	140.12	138.76	160.29	186.58	204.31	203.50
70–74	135.44	147.79	170.44	195.56	201.40	223.00	253.46
75–79	145.41	178.22	186.70	226.86	243.87	234.63	263.24
80–84	123.63	160.33	175.09	229.23	267.55	264.61	276.39
85+	133.97	232.56	212.33	227.57	248.07	292.51	272.51
Females							
40–44	6.75	6.14	4.90	5.11	3.46	2.88	3.03
45–49	11.99	11.10	9.21	9.76	6.49	5.76	4.90
50–54	18.20	16.23	14.78	15.53	14.15	11.78	10.20
55–59	27.57	30.06	24.45	28.43	28.65	28.04	23.68
60–64	35.23	39.48	41.09	44.75	55.75	52.97	48.01
65–69	43.49	54.68	52.09	66.36	76.92	89.05	82.80
70–74	50.40	61.49	65.48	87.47	111.46	130.60	131.32
75–79	66.47	72.02	77.57	105.09	127.37	153.26	160.98
80–84	60.12	74.79	81.83	101.26	139.41	175.41	184.90
85+	54.22	82.91	71.93	103.98	132.42	169.12	196.03

Let the mortality rate of the *i* ^th^ age group and the *j* ^th^ period group be denoted by *λ_ij_* The APC model is as follows:

$$\begin{array}{cc} \log\;\lambda_{ij}=\mu +\alpha_{i}+\beta_{j}+\gamma_{k,} & \;i=1,\;2,...,I,\\ & j=\;1,2,...,J,\;\\ & k=j-i+I, \end{array}$$ (Eq-1)

where the intercept term is represented by *μ*, the age effects by *α_i_*, the period effects by *β_j_*, and the cohort effects by *γ_K_*. The following constraints are used:

$$\sum_{i}\alpha_{i}=\sum_{j}\beta_{j}=\sum_{k}\gamma_{k}=0$$

### The multiphase method in estimating cohort effects

The multiphase method includes three-phase processes that concretized the estimation of the cohort effect as a partial interaction in age-period contingency table data [6, 9]. The natural log rate (*λ_ij_*) is established using the log-additive effect as a constant term plus age, period effect, and multiplicative interaction term, which can be regarded as a fully saturated model that includes systematic and unsystematic components (random error). The systematic component contains cohort effect. After isolating the residuals from the median polish phase, the final phase is to estimate the magnitude of cohort effects through regression of these residuals (*Ɛ_k_*) on the cohort category (cohort is an indicator variable entered as a collection of the m + n–1 cohorts as k = 1,2,…, m + n–1)

$$\varepsilon_{k}=\gamma_{k}+\varepsilon_{ijk}$$ (Eq-2)

The *Ɛ_k_* is established using a vector of cohort effects (*γ_k_*) and error terms (*Ɛ_ijk_*), where *Ɛ_ijk_* represented the error terms unmeasured as *i* age, *j* period, and *k* cohort categories.

According to the cohort-specific mortality by age that calculated as removed and unremoved cohort influence to decide reference categories. Reference categories of cohort had a minimum difference in cohort-specific mortality between with and without cohort influence. After subtracting residuals form contingency data, we can use the residual to calculate the log-additive rate (without cohort effect) with multiplying factor *e*^–(residual)^ to the rate each age and period group. Then, take the ratio of log-additive rate without and with cohort effects for each cohort. If the rate ratio of the cohort is close to one, then it is determined as the referent birth cohort. The referent birth cohort can be determined based on the slight variation of its rate after removing the influencing factors.

### Weighted average

For birth cohorts with members that achieved the cohort, let *W_k_* denote the weight of the *k* ^th^ cohort category:

$$\varepsilon_{k}=W_{k}\times \gamma_{k}+\varepsilon_{ijk}$$ (Eq-3)

However, the common assumption is that each cohort across *i* age and *j* period of data provides equal information (i.e., equally weighted) for the estimation of cohort effects in a model while the weighting factor is generally unknown. Empirically, the equally weighted assumption is usually violated while modeling to estimate the cohort effect. The empirical weighting factor most widely used is the number of death [20]. Each of these weights can be applied to the regression equation. The weighted average of the cohort effect can be performed via the weight equal to the occupied proportion according to the number of deaths due to HCC in each cohort.

To check model fitness and furthermore we plot deviance residuals which from the null model, the age model, the age-period model, and then to the APC model (under the proposed weighted method) progressively (Supplementary Figure 1).

## SUPPLEMENTARY MATERIALS FIGURE


