# Supplementary material for: Using weighted regression model for estimating cohort effect in age-period contingency table data
Source: Oncotarget. 2018 Apr 13;9(28):19826–35. doi: 10.18632/oncotarget.24868 (PMC5929429; doi:10.18632/oncotarget.24868)
Supplement: Supplementary file 1 [file oncotarget-09-19826-s001.pdf]

Using weighted regression model for estimating cohort effect in age-period contingency table data

SUPPLEMENTARY MATERIALS

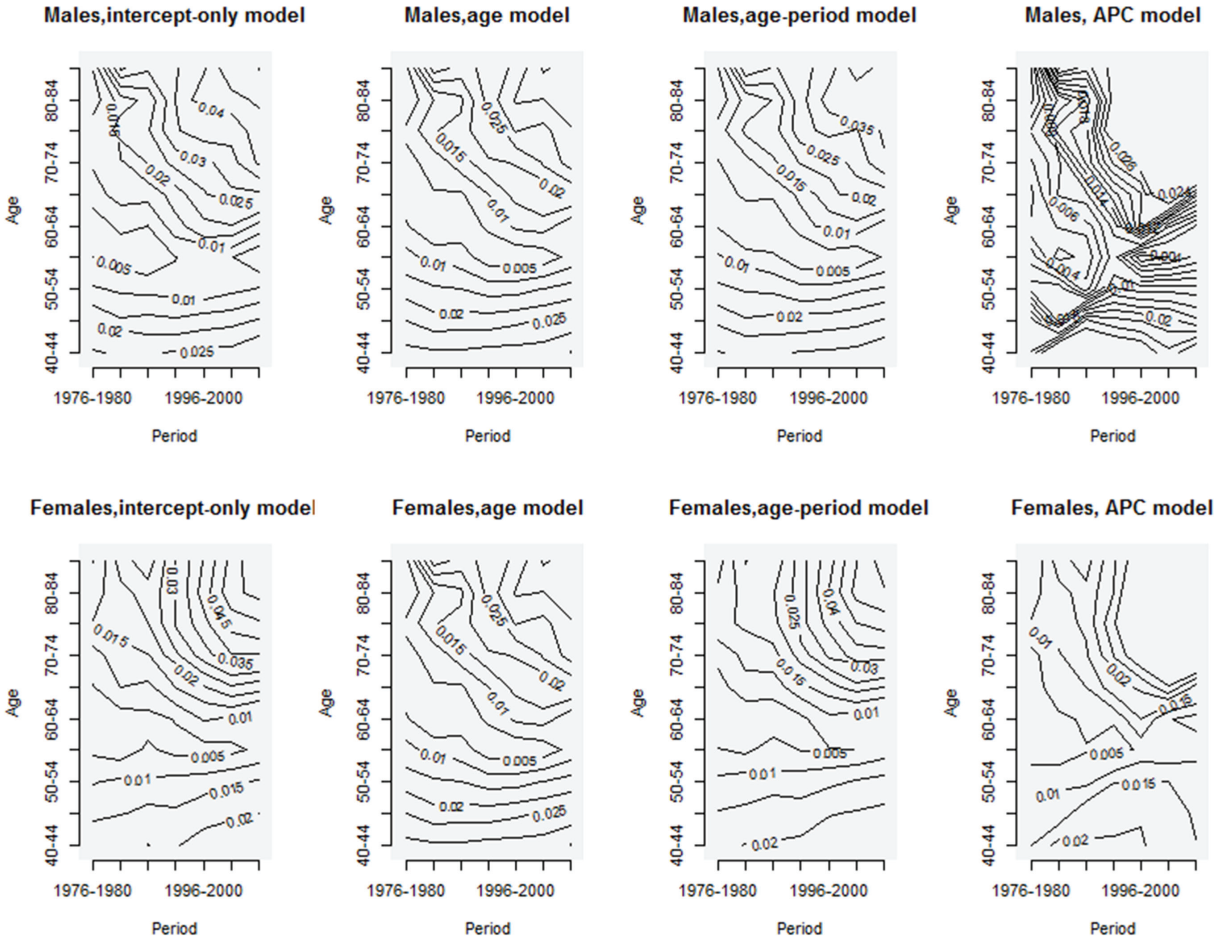

Supplementary Figure 1: Residual plots for the null models, the age models, the age-period (AP) models, and the age-period cohort (APC) models.
